# Coronavirus Anxiety Level and COVID-19 Vaccine Attitude Among Patients With Hematological Malignancies

**DOI:** 10.7759/cureus.38618

**Published:** 2023-05-06

**Authors:** Zeynep Tuğba Güven, Serhat Çelik, Muzaffer Keklik, Ali Ünal

**Affiliations:** 1 Hematology, Adana City Hospital, Adana, TUR; 2 Hematology, Kırıkkale University School of Medicine, Kırıkkale, TUR; 3 Hematology, Erciyes University School of Medicine, Kayseri, TUR

**Keywords:** coronavirus disease, covid 19, vaccination attitudes, coronavirus anxiety scale, sars-cov-2, hematological malignancy, covid-19 vaccination

## Abstract

Introduction: The COVID-19 vaccine is the most essential tool for altering the pandemic's trajectory. The pandemic's control is complicated by society's unwillingness to vaccinate. The aim of this cross-sectional study was to assess patients with hematological malignancies and their attitudes regarding COVID-19 immunization and to investigate COVID-19 anxiety in this susceptible population.

Methods: In this cross-sectional study, 165 patients with hematological malignancies were included. COVID-19 anxiety was evaluated with the coronavirus anxiety scale (CAS), and COVID-19 vaccine attitude was evaluated with the Vaccine Attitudes Review (VAX) scale.

Results: The mean CAS score was 2.42 (0-17). There were 22 (13%) participants with a mean CAS score of ≥ 9. Half of the participants had a CAS score of 0. The CAS score was higher in females (p = 0.023). Similarly, it was significantly higher in patients who were not in remission for hematological malignancy and who received active chemotherapy (p = 0.010). The mean VAX score was 49.07 ± 8.76 (27-72). Most of the participants (64%) had a neutral attitude toward the COVID-19 vaccination. In a survey of 165 patients, 55% said that they were skeptical about vaccination safety, and 58% said that they were concerned about unintended side effects. In addition, 90% expressed moderate concerns about commercial profiteering. Natural immunity was preferred by 30% of the participants. There was no statistically significant correlation between CAS scores and the Vaccine Attitudes Review (VAX) scale.

Conclusion: This study draws attention to the level of anxiety in patients with hematological malignancies during the COVID-19 pandemic. Negative attitudes toward the COVID-19 vaccine are worrisome for at-risk patient groups. We think that patients with hematological malignancies should be informed to eliminate their hesitations about COVID-19 vaccines.

## Introduction

The COVID-19 virus was detected in December 2019 and spread extensively over the world; the World Health Organization designated it a pandemic in March 2020 [1]. From the start of the pandemic to March 6, 2022, more than six million deaths were reported [2]. Many studies have demonstrated that COVID-19 is related to higher mortality rates in patients with cancer [3-6].

There is a lot of negative news about the pandemic on the internet, television, and social media, often including the number of cases and deaths. This causes the development of coronaphobia in society [7]. Similarly, the prevalence of fear, anxiety, and depression associated with COVID-19 is high in cancer patients [8-10]. One of the best public health measures for preventing and controlling infectious diseases is vaccination [11]. Due to concerns about the safety of vaccines developed against a new virus, the possibility of side effects, and uncertainty about protection, individuals are cautious about vaccines [12]. Vaccine hesitancy is the delay in accepting a vaccine or the rejection of a vaccine despite its availability. This is an increasing threat to both national and global health [13].

It has been shown in studies that patients, especially those with hematological malignancies, should be evaluated for COVID-19 vaccination in very high-risk categories and should be vaccinated primarily [14]. There are very few studies that determine the attitudes of patients with hematological malignancies toward COVID-19 vaccines.

This study's objective was to assess the attitudes of patients with hematological malignancies towards vaccination and determine the relationships between vaccination hesitancy and patient characteristics. The secondary aim was to identify the pandemic-related anxiety level of this patient group and to investigate whether anxiety influences vaccination propensity.

This article was previously presented as a meeting abstract at the XIII Eurasian Hematology-Oncology Congress in Istanbul, Turkey, on October 6, 2022.

## Materials and methods

Population data

This cross-sectional study was conducted with hematologically malignant patients at the Hematology Clinic of the Erciyes University Hospital, in Kayseri, Turkey, from May 1 to December 1, 2021. Patients who (1) were at least 18 years old, (2) willingly accepted to participate in the survey, and (3) could understand and answer the questionnaire met the inclusion criteria.

Consent and ethical approval

Patients were briefed about the study's purpose and outcomes, and after all of their questions were answered, they signed a written informed consent form. The study protocol was reviewed and approved by the Institutional Review Board at Erciyes University. The Erciyes University Scientific Ethics Committee approved the study on September 22, 2021, with approval number 2021/625.

Research instruments

The questionnaire consisted of three parts. In the first section of the study, questions regarding the patients' sociodemographic characteristics, including age, gender, diagnosis, disease, hematopoietic stem cell transplantation (HSCT) history, educational status, marital status, and place of residence, were asked. Participants' histories of vaccinations and COVID-19 infection status were questioned on a yes-or-no basis. Two questions were used to assess previous vaccination histories: "Have you ever been vaccinated against seasonal flu in the last two years?" and "Have you ever been vaccinated against COVID-19?"

Coronavirus anxiety scale (CAS)

The second instrument was the COVID-19 pandemic-related anxiety scale or coronavirus anxiety scale (CAS) [15], which is standardized. The five items on the coronavirus anxiety scale range from 0 (not at all) to four (nearly every day over the last two weeks) on a five-point Likert-type scale. An individual was classified as having anxiety if their score was nine or greater. The CAS has good diagnostic viability and construct validity. The validity and reliability study of the scale's Turkish version was carried out by Evren et al. The scale's internal dependability was good (Cronbach's alpha = 0.80) [16].

Vaccination attitudes examination scale (VAX)

The 12-item Vaccine Attitudes Review (VAX) scale [17] was employed in the third section to assess general attitudes regarding vaccines. "Mistrust of vaccine benefits," "concerns about unknown future effects," "concerns about commercial profiteering," and "desire for innate immunity" are the four subscales on this scale. While answering the questions, participants were instructed to concentrate on the COVID-19 vaccination. The responses were graded on a six-point Likert scale ranging from one to six, with one indicating "strongly agree" and six indicating "strongly disagree." The scores for each subscale of the VAX instrument were categorized into three levels of negative attitudes toward vaccines: high (score of five to six), intermediate (score of three to four), and low (score of one to two). A higher VAX total score indicates a higher intensity of anti-vaccination attitudes. Yıldız et al. conducted content validity and reliability research in Turkey. R = 0.818 was the Cronbach alpha coefficient [18].

Statistical analysis

The IBM Statistical Package for Social Sciences (SPSS) was used to examine the study's data (version 25 for Windows, IBM Corp., Armonk, NY, USA). When using the mean and standard deviation for continuous data, absolute and percentage values were used for categorical variables. To evaluate the parameters' distribution, the Shapiro-Wilk test was used. Chi-square tests were used to make statistical comparisons on categorical data. Continuous numerical data were compared using the Student t-test for two independent samples. The relationship among quantitative data was evaluated using the Spearman correlation analysis. Values of p<0.05 were considered to indicate statistical significance.

## Results

Characteristics of the study group

The basic demographics of the study group are shown in Table 1.

**Table 1:** Demographic and clinical characteristics of the study group (n = 165)

	Descriptive statistics n (%)
Age (in years)	48 (18 - 86)
18 – 39	53 (32)
40-59	62 (38)
>60	50 (30)
Gender, n (%)	
Female	61 (37)
Male	104 (63)
Diagnosis, n (%)	
Acute myeloid leukemia	61 (37)
Acute lymphoblastic leukemia	14 (9)
Multiple myeloma	22 (13)
Hodgkin's lymphoma	15 (9)
Non-Hodgkin's lymphoma	49 (30)
Other	4 (2)
Hematopoietic stem cell transplantation, n (%)	
No	120 (73)
Yes	45 (27)
Autologous	18 (11)
Allogeneic	27 (16)
Disease status, n (%)	
Active	117(71)
Remission	48(29)
Comorbidities, n (%)	
No	130(79)
Yes	35(21)
Education level, n (%)	
Primary school	61 (37)
Middle school	33 (20)
High school	38 (23)
University	33 (20)
Marital status, n (%)	
Married	130 (79)
Unmarried	35 (821)
Place of residence, n (%)	
Rural	49 (30)
Urban	116 (70)
Have you ever tested positive for COVID-19? n (%)	
No	116 (70)
Yes	49 (30)
Have you ever been vaccinated against COVID-19? n (%)	
No	93 (56)
Yes	72 (44)
Have you ever been vaccinated against seasonal flu in the last two years? n (%)	
No	128 (77)
Yes	37 (23)

After removing those who did not complete the questionnaires (n = 12), the study comprised a total of 165 participants. The median age was 48 (18-86) years, 61 (37%) of whom were female. Most of the participants (37%) were diagnosed with acute myeloid leukemia and were undergoing chemotherapy. In addition, 21% of the patients reported having comorbidities. For educational levels, 37% (n = 61) attended primary school, 20% (n = 33) had middle school instruction, 23% (n = 38) received high school education, and 20% (n = 33) obtained university education. In terms of marital status, 79% (n = 130) of the patients were married. When the participants were evaluated according to their place of residence, 70% (n = 116) lived in urban areas and 30% (n = 49) lived in rural areas. At the time of the survey, 70% of the patients had not been infected with COVID-19, whereas 44% had been vaccinated. Furthermore, 23% had been vaccinated for the seasonal flu in the previous two years.

Outcomes of VAX

**Figure 1:**
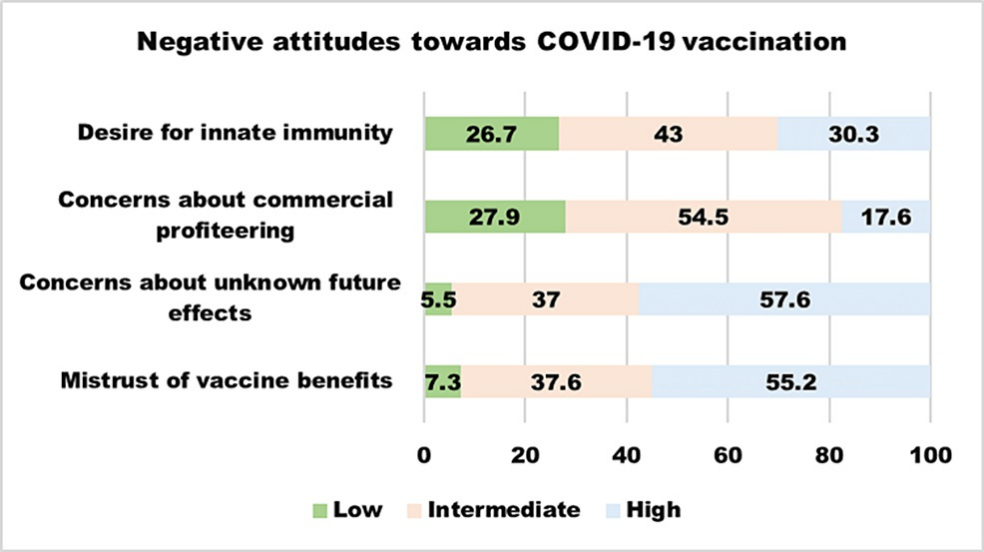
A proportion of the sample reporting high, intermediate, and low negative attitudes towards vaccines

The mean VAX score was 49.07 ± 8.76 (27-72). Most of the participants (68%) had a neutral attitude towards the COVID-19 vaccination. Of the 165 participants, 55% reported high distrust of vaccine safety, while 38% were unsure about their level of confidence. Furthermore, 58% reported severe concerns about unexpected adverse effects, while 37% reported moderate concerns. Additionally, 18% expressed serious doubts about commercial profiteering. Natural immunity was highly desired by around 30% of the participants, whereas natural immunity was thought to be superior to vaccination by 43% (Figure 1).

The average of the three items each in the "mistrust of vaccine benefit," "concerns about unknown future effects", "concerns about commercial profiteering", and "desire for innate immunity" subscales were used as the outcome measure, and the average participant scores were 4.66, 4.72, 3.2, and 3.75, respectively. The results are recorded in Table 2.

**Table 2:** Scores of the coronavirus anxiety scale (CAS) and Vaccination Attitudes Examination (VAX) scale

Variables	Mean / Standard deviation / Range	Items
CAS	2.42 ± 3.46 (0-17)	1-5
VAX	49.07± 8.76 (27-72)	1-12
Mistrust of vaccine benefits	4.66 ± 1.26	1-3
Concerns about unknown future effects	4.72±1.19	4-6
Concerns about commercial profiteering	3.22±1.24	7-9
Desire for innate immunity	3.75±1.54	10-12
Total	4.08±0.73	1-12

Outcomes of CAS

The sample’s overall CAS mean score was 2.42 (SD = 3.47) (0-17). There were 22 (13%) participants with a mean CAS score of ≥ 9. Half of the participants had a CAS score of 0. Eighty-two (50%) patients had some level of anxiety (CAS score 1-20). By gender, women expressed a higher CAS score than men (p = 0.023). The mean score for women on the CAS was 3.39, while the mean score for men was 1.85. Similarly, it was significantly higher in patients who were not in remission for a hematological malignancy and who had received active chemotherapy (p=0.010) (Table 3).

**Table 3:** Investigation of the relationship between demographic and clinical characteristics and CAS and VAX scores

Variables	CAS	p-value	VAX	p-value
Age (in years)				
18 – 39	1.58±3.26	0.079	50.7 ±9.18	0.174
40-59	2.82±3.74		48.9 ±7.69	
>60	2.80±3.22		47.52 ±9.39	
Gender				
Female	3.39±4.08	0.023	50.6±8.60	0.073
Male	1.85±2.82		48.14±8.75	
Diagnosis				
Acute myeloid leukemia	2.64±3.49	0.894	49.83±7.93	0.898
Acute lymphoblastic leukemia	1.71±2.73		50.85±8.55	
Multiple myeloma	1.55±2.87		47.53±11.45	
Hodgkin's lymphoma	2.27±4.35		47.68±7.10	
Non-Hodgkin's lymphoma	2.86±2.87		48.6±9.52	
Other	1.62±2.63		45.7±10.37	
Disease status				
Active	2.83±3.67	0.010	48.41±8.65	0.131
Remission	1.42±2.68		50.68±8.88	
Comorbidities				
Yes	2.86±3.11	0.108	47.80±8.43	0.563
No	2.30±3.55		49.42±8.85	
Hematopoietic stem cell transplantation				
Yes	1.87±3.2	0.086	49.22±8.76	0.963
No	2.63±3.52		49.02±8.80	
Education level		0.115		0.699
Primary school	3.41±3.95		48.91±8.42	
Middle school	2.03±2.54		48±10.18	
High school	1.74±2.91		50.4±7.26	
University	1.76±3.59		48.8±9.59	
Marital status				
Unmarried	1.91±3.64	0.160	51±10.53	0.051
Married	2.55±3.41		48.56±8.19	
Place of residence				
Rural	2.51±3.44	0.645	49.51±8.62	0.68
Urban	2.38±3.49		48.89±8.84	
Have you ever tested positive for COVID-19?				
Yes	2.8±3.95	0.872	50.28±9.32	0.251
No	2.26±3.24		48.56±8.50	
Have you ever been vaccinated against COVID-19?				
Yes	2.72±3.74	0.232	47.69±8.71	0.074
No	2.18±3.23		50.15±8.69	

Evaluation of CAS relationship with the VAX scale

There was no statistically significant correlation between CAS scores and the Vaccine Attitudes Review (VAX) scale (Table 4).

**Table 4:** Investigation of the correlation between CAS scores and the Vaccine Attitudes Review (VAX) scale

VAX	CAS>9	Cas<9	p-value
Mistrust of vaccine benefit (items 1–3)			
Low	1 (0.6)	11 (7)	0.404
İntermediate	6 (3.6)	56 (34)	
High	15 (9)	76 (46)	
Concerns about unknown future effects (items 4-6)			
Low	1 (0.6)	8 (5)	0.974
İntermediate	8 (4.8)	53 (32)	
High	13 (8)	82 (50)	
Concerns about commercial profiteering (items 7-9)			
Low	5 (3)	41(24.8)	0.649
Intermediate	14 (8.5)	76 (46)	
High	3 (1.8)	26(15.8)	
Desire for innate immunity (items 10–12)			
Low	4 (2.4)	40 (24.2)	0.591
İntermediate	11 (6.7)	60 (36.4)	
High	7 (4.2)	43 (26.1)	
Total (items 1–12)			
Low	0 (0)	2 (1.2)	0.748
İntermediate	15 (9.1)	97 (58.8)	
High	7 (4.2)	44 (26.7)	

## Discussion

Our study, to our knowledge, is the first to assess how patients with hematological malignancies think about COVID-19 vaccines and their anxiety associated with COVID-19. When we reviewed the literature, there was a gap in studies evaluating COVID-19 anxiety and attitudes toward vaccines in patients with hematological malignancies. This included a large number of patients with hematological malignancies, representing a vulnerable population most affected by the pandemic. According to research conducted on cancer patients, COVID-19 caused them great dread and distress [10, 19-21]. In our study, it was found that the mean CAS score of the patients was 2.42 ± 3.47. Half of the participants had a CAS score of 0. While conducting our study, we expected a high level of COVID-19-related anxiety in patients. However, we found low anxiety scores in our study. Our findings were not compatible with the literature's findings. Studies have shown that a high level of knowledge among cancer patients is associated with decreased anxiety [22].

Since the beginning of the pandemic, we have informed our patients in detail about COVID-19 and tried to address their concerns. We think that our patients have a high level of knowledge about the COVID-19 infection and its management. Anxiety was significantly higher in female patients and those with active disease. In a study analyzing the psychological distress of lymphoma patients during the COVID-19 pandemic, women were revealed to be at higher risk, which is similar to our data. [23]. This may be due to cultural differences. In Turkish society, men may not express their feelings as much as women. In a study of cancer patients, 34.3% of patients rated cancer as more dangerous than COVID-19, while 59% thought that COVID-19 was more severe in cancer patients. In the same study, 45.6% of the patients reported that the pandemic negatively affected their cancer care [24].

In our study, a statistically higher rate of anxiety was found in cancer patients receiving active chemotherapy than in patients in remission. The COVID-19 pandemic has caused problems, such as not being able to receive treatment due to the lack of health resources among individuals, who have to apply to the hospital regularly for follow-up and treatment. In addition, the thought that there may be a delay in diagnosis and treatment for cancer patients receiving active treatment may have created more anxiety.

This study showed an intermediate vaccine hesitancy score (M = 4.08). In a study of 3960 people who expressed their hesitation about the COVID-19 vaccine, the most frequently expressed reason was concerns about the novelty of the vaccine. At the same time, general distrust was widespread due to concerns about the safety of the vaccine [25]. Our findings suggest that patients with hematological malignancies have a distrust of the benefits of COVID-19 vaccines and concerns about their unpredictable effects. Studies have shown that the groups with the highest mistrust of vaccines are women, children, and individuals with low education levels and low socioeconomic status [26-30]. We found no association between demographic and clinical characteristics and vaccination acceptance.

However, it provides important data for evaluating factors associated with vaccine hesitancy in patients with hematological malignancies. Vaccination is the most important strategy in the fight against COVID-19. Emphasizing the importance of vaccination, especially in vulnerable patient groups, may facilitate the acceptance of COVID-19 vaccines.

Due to its cross-sectional design and small sample size, this study has some limitations. Our study's lack of a control group made up of healthy adults is another drawback.

## Conclusions

This study showed that patients with active cancer have a relatively higher rate of COVID-19 anxiety. Our findings are important in terms of demonstrating the mistrust of the usefulness of the vaccine in patients with hematological malignancies, concerns about side effects, and commercial concerns. One of the biggest barriers to vaccination is misinformation, especially on the Internet. Increasing knowledge about vaccination and promoting trust among health authorities can help reduce vaccination hesitancy. Hematologists and oncologists should promote the COVID-19 vaccine for patients with cancer.
